# Neither early nor late for becoming pregnant: Comparison of the perinatal outcomes of adolescent, reproductive age, and advanced maternal age pregnancies

**DOI:** 10.4274/tjod.94758

**Published:** 2015-09-15

**Authors:** Orkun Çetin, Fatma Ferda Verit, Ali Galip Zebitay, Zuhal Aydın, Zehra Kurdoğlu, Oğuz Yücel

**Affiliations:** 1 Yüzüncü Yıl University Faculty of Medicine, Department of Obstetrics and Gynecology, Van, Turkey; 2 Süleymaniye Education and Research Hospital, Clinic of Obstetrics and Gynecology, İstanbul, Turkey; 3 Ankara Education and Research Hospital, Clinic of Obstetrics and Gynecology, Ankara, Turkey; 4 Adana Numune Education and Research Hospital, Clinic of Obstetrics and Gynecology, Adana, Turkey

**Keywords:** Age maternal, Pregnancy, outcome

## Abstract

**Objective:**

To compare perinatal and short-term neonatal outcomes of adolescent, reproductive age, and advanced maternal age (AMA) pregnancies in a low-income region of İstanbul.

**Materials and Methods:**

Three hundred six adolescents, 301 reproductive age, and 303 AMA pregnant women who delivered in Süleymaniye Education and Research Hospital between January 1^st^ 2007, and January 31^st^ 2015, were recruited to the study population. The clinical, obstetric and short-term neonatal outcomes of the women were analyzed retrospectively.

**Results:**

Adolescent and AMA pregnancies were associated with severe adverse perinatal and short-term neonatal outcomes compared with reproductive-age women. Adolescent and AMA pregnancies had quite similar risks in obstetric outcomes. Adolescent pregnancies were related with severe adverse short-term neonatal outcomes when compared with advanced maternal age pregnancies.

**Conclusion:**

Adolescent and AMA pregnancies should be defined as high-risk pregnancies. Our research indicated that healthcare providers such as obstetricians, midwives, and family physicians should be alert in these populations.

## INTRODUCTION

The effect of maternal age as a risk factor for pregnancy has been well studied. Having a baby in early or late years of a woman’s life may be related to adverse perinatal outcomes^(1)^. Adolescent pregnancy was described by the World Health Organization as pregnancy in girls aged between 10-19 years^(2)^. Although it is a substantial health and social matter around the world,^(3)^ most cases were seen in developing countries and carry remarkable risk^(2)^. Biologic immaturity, unwanted pregnancy, insufficient prenatal care, deficient maternal diet, maternal stress, poverty, low education, deficient parent support, inadequate weight gain, and anemia may cause adverse pregnancy outcomes in adolescent pregnancies^(4,5)^.

In the last few decades, delayed childbearing is a growing tendency in developing countries^(6)^. Although the relationship towards advanced maternal age (AMA), fetal anomaly rate, and diminished fecundity has been well demonstrated, previous reports about perinatal outcomes of AMA were controversial^(1,7)^. A previous study indicated that deficient uterine vascularity associated with decreased fetal oxygenation and increased risk of hypertension in older women were the main reasons for adverse perinatal outcomes in AMA^(8)^.

A recent study represented a mean age-specific fertility rate of 35 per 1000 women aged between 15-19 years. The adolescent pregnancy rate was found as 6.3% across Turkey and 3.92% in İstanbul according to the Turkish Statistical Institute (2014) data^(9)^. Rates of adolescent childbirth varies considerably between the regions of Turkey. Furthermore, the AMA pregnancy rate was 2.75% across Turkey and 2.65% in İstanbul^(9)^. However, epidemiologic research on AMA pregnancies in Turkey is limited.

The aim of the present study was to compare perinatal and short-term neonatal outcomes of adolescent, reproductive age, and AMA pregnancies in a low-income region of İstanbul.

## MATERIALS AND METHODS

An observational and case-control study was performed to investigate the perinatal outcomes of adolescent, AMA, and reproductive-age women in an education and research hospital in a low-income region of İstanbul. The sample size was determined by 95% probability and 90% power. According to the formula, the minimum number of pregnant women was calculated as 280 for each group. The study population was divided into three groups that consisted of 348 adolescents (group 1), 301 reproductive aged (group 2), and 340 AMA (group 3) pregnant women who delivered in Süleymaniye Education and Research Hospital between January 1^st^ 2007, and January 31^st^, 2015. Clinical data were retrospectively documented using the computerized medical records system of Süleymaniye Education and Research Hospital. Missing clinical data were tracked and completed using patient files.

For each group, we recorded clinical and demographic information of the enrolled patients including maternal age at delivery, gravidity, parity, multiple gestation, cigarette smoking status, history of fetal death, history of preterm birth, assisted reproductive technology (ART), congenital abnormalities, anemia, urinary infection, gestational diabetes mellitus (GDM), and gestational hypertension (GHT). Perinatal outcome data comprised gestational week at delivery, mode of delivery, preeclampsia, eclampsia, preterm birth, placenta previa, placental abruption, preterm premature rupture of membranes (PPROM), stillbirth, fetal distress, shoulder dystocia, retained placenta, atonia, perineal injury and hospitalization time. Neonatal outcome data consisted of birth weight, low birth weight (LBW), first and fifth minute Apgar scores, respiratory distress syndrome (RDS), neonatal sepsis, hyperbilirubinemia, necrotizing enterocolitis (NEC), convulsions, hypoglycemia, hypocalcemia, and neonatal intensive care unit (NICU) admission for each study group.

AMA was defined as mothers aged 40 years or older at the time of delivery. Adolescent pregnancy was defined as mothers aged 18 years or younger at the time of delivery. Reproductive woman was defined as a mother aged between 18-40 years at the time of delivery. The pregnant women at reproductive age were chosen randomly using a computer from 4157 women who delivered in Süleymaniye Education and Research Hospital during the defined study period. Randomization was made using a ‘‘random numbers’’ table. Stillbirth was described as fetal death at or after 24 weeks gestation. Preeclampsia and GHT were diagnosed with the guidelines of the International Society for the Study of Hypertension in Pregnancy^(10)^. Gestational hypertension was described as systolic blood pressure ≥140 mmHg and/or diastolic blood pressure ≥90 mmHg on at least two occasions 4 hours apart, appearing after 20 weeks gestation in normotensive women in the lack of proteinuria. Preeclampsia was described as gestational hypertension with proteinuria of ≥300 mg in 24 hours. The diagnosis of GDM was made with double-step screening. An oral glucose challenge test (50 g) was performed at 24-28 weeks gestation among all pregnant women if the concentration was ≥140 mg/dL in the first hour; 100 g oral glucose tolerance test was performed after 2 weeks. GDM was diagnosed if the two values were higher than normal ranges (normal ranges; 0 hours: 95 mg/dlL, 1^st^ hour: 180 mg/dL, 2^nd^ hour: 155 mg/dL, 3^rd^ hour: 140 mg/dL). Preterm delivery was described as labor before 37 weeks of gestation. The lower limit of preterm delivery was 28 weeks of gestation. Spontaneous preterm delivery included spontaneous commencement of labor and PPROM. Iatrogenic preterm delivery indications were preeclampsia, intrauterine growth restriction, placental abruption, placenta previa, fetal distress, and other obstetric complications. LBW neonates were described as birth weight below 2500 g. Emergency cesarean section indications included failure to progress labor, fetal distress, or hemorrhage during delivery. Forty-two adolescent and 37 AMA pregnant women were excluded from the study because of incomplete perinatal data.

The study was conducted in accordance with the declaration of Helsinki and was approved by the local ethics committee of Süleymaniye Education and Research Hospital Planning Committee.

### Statistical analysis

Categorical variables were described as number (n) and percent (%), while continuous variables were expressed as mean ± standard deviation, confidence interval (95%), and minimum and maximum values. Continuous variables were compared among the three groups using one-way analysis of variance (ANOVA) test. A Chi-square test was used to examine the association between categorical variables. Duncan’s test was performed to determine which group differed significantly from other groups. P values of <0.05 were considered to be significant. Statistical analysis was performed using Statistical Package for Social Sciences for Windows (version 20.0) software.

## RESULTS

The clinical characteristics of the study population are given in Table 1. In total, 910 pregnant women were recruited who fulfilled the inclusion criteria: 306 adolescents, 301 reproductive aged, and 303 AMA pregnant women. The youngest patient was aged 13 years and the oldest was aged 47 years. The mean maternal age was 15.78±0.89 years for group 1, 26.59±4.2 years for group 2, and 40.93±0.95 years for group 3. Three hundred one patients (98.4%) in group 1, 19 (6.3%) patients in group 2, and 26 (8.6%) patients in group 3 were nulliparous. Significant differences were found in the rates of multiple gestation (p=0.001), cigarette smokers (p=0.034), history of fetal death (p=0.001), history of preterm birth (p=0.001), ART (p=0.001), anemia (p=0.001), urinary infection (p=0.001), and GHT (p=0.001) between all groups (Table 1). The fetal abnormality rate was similar in each group. AMA pregnancies were characterized by a significantly higher risk of multiple gestation (Odds ratio (OR): 2.19, 95% Confidence interval (CI): 1.13-4.26), history of fetal death (OR: 35.34, 95% CI: 4.87-256.37), history of preterm birth (p=0.001), ART pregnancies (p=0.001), GDM (OR: 3.60, 95% CI: 2.12-6.12) and GHT (OR: 2.07, 95% CI: 1.23-3.50) compared with adolescent pregnancies. Adolescent mothers were more likely to have anemia than AMA mothers (p=0.016). Rates of fetal abnormalities, urinary infections, and cigarette smoking were similar between group 1 and group 3.

The obstetric outcomes of each group are listed in Table 2. The mean gestational week at delivery was 34.26±3.86 for group 1, 36.73±3.31 for group 2, and 35.90±3.10 for group 3. There were significant differences between the three groups in duration of pregnancy (p=0.001). Moreover, there were significant differences between the three groups in the rates of vaginal delivery, cesarean section, preeclampsia, preterm birth, placenta previa, stillbirth, fetal distress, perineal injury, and hospitalization time. There were no significant differences in eclampsia, placental abruption, PPROM, shoulder dystocia, retained placenta, and atonia (Table 2). AMA pregnancies had significantly longer duration of gestation compared with adolescent gestations (p=0.001) (Table 2). The rates of vaginal delivery, cesarean section, preeclampsia, eclampsia, placental abruption, PPROM, stillbirth, shoulder dystocia, retained placenta, atonia, perineal injury, hospitalization time, and emergency cesarean section were similar among adolescent and AMA pregnancies. Preterm birth was more common in group 1 than in group 3 (p=0.001). The rate of placenta previa was higher in group 3 than in group 1 (p=0.001). Fetal distress and perineal injury rates were significantly higher in the adolescent group (p=0.002 and p=0.001, respectively).

Table 3 summarizes the short-term neonatal outcomes of each group. There were significant differences between groups in birth weight (p=0.001), Apgar score at minute 1 (p=0.001), Apgar score at minute 5 (p=0.001), and rates of LBW (p=0.001), RDS (p=0.004), neonatal sepsis (p=0.048), hyperbilirubinemia (p=0.001), hypocalcemia (p=0.004), and NICU admission (p=0.001). Newborns in group 1 had lower birth weight than the other groups. Newborns in group 2 had higher Apgar scores at the 1st and 5th minutes than others. The rate of NEC was similar between all 3 groups (p=0.061) (Table 3). Adolescent pregnancies were characterized by significantly higher risks of LBW (OR: 1.897, 95% CI: 1.31-2.74); neonatal sepsis (p=0.014); NEC (p=0.014); convulsions (OR: 6.993, 95% CI: 2.06-23.79); hyperbilirubinemia (OR: 1.809, 95% CI: 1.19-2.76); hypoglycemia (OR: 10.870, 95% CI: 3.28-36.02); hypocalcemia (OR: 3.729, 95% CI: 1.03-13.50); and NICU admission (OR: 3.365, 95% CI: 1.90-5.98) compared with AMA pregnancies.

## DISCUSSION

The main consequences of the current study were that both adolescent and AMA pregnancies were associated with severe adverse perinatal and short-term neonatal outcomes compared with reproductive-age women. Adolescent and AMA pregnancies had similar risks in obstetric outcomes. Furthermore, adolescent pregnancies were related with severe adverse short-term neonatal outcomes compared with AMA pregnancies. Although perinatal outcomes of adolescent and AMA pregnancies have been reported for each group in many previous studies, few quantitative studies have compared perinatal outcomes of adolescent, reproductive age, and AMA pregnancies together.

Adolescent pregnancy is an increasing health problem worldwide^(2)^. On the other hand, in recent years, many couples in industrialized countries have preferred to delay marriage and childbearing. Pregnancies at early or late maternal ages are both associated with increased rates of obstetric and perinatal complications^(3,11)^.

An increased risk of maternal anemia was found in AMA pregnancies^(12)^. In the present study, anemia was more common in adolescent pregnancies as in previous studies^(13)^. Additionally, the risk of anemia for adolescent pregnancies is 1.58-fold greater than AMA pregnancies. Recent studies concluded that inadequate prenatal care was the main reason of anemia in adolescent pregnancies^(2,13,14,15)^. Our study design did not verify prenatal care visits of pregnancies; however, it is our consideration that lifestyle, adequate prenatal visits, and socioeconomic conditions of pregnant women may be crucial for maternal anemia.

The increased risk for fetal congenital abnormalities with AMA is documented in previous studies^(7,16)^. However, the incidence of congenital abnormalities was similar between each group in our study population. Consistent with our study, Weerasekera et al.^(17)^ reported a similar result in Sri Lanka. Advanced age has an adverse impact on reproductivity,^(7)^ and as such, pregnancies with ART were more common in the AMA group. Consequently, the multiple gestation rate was higher in AMA pregnancies in our study.

Older pregnant women may have extra comorbidities such as hypertensive disorders and diabetes mellitus^(11)^. In the present study, GDM and gestational hypertension were more common in the AMA group than in the other groups. Additionally, the risk of GDM for AMA pregnancies was 3.60-fold higher than in adolescent pregnancies. Also, the risk of gestational hypertension for AMA was 2.07-fold higher than in adolescent mothers.

Low rates of cesarean deliveries and higher proportions of vaginal deliveries were defined in adolescent pregnancies^(20,21,22)^. However, we found a significant increase in cesarean delivery rates for adolescence compared with reproductive-age women, inconsistent with the prior studies. We explained this result with the higher proportions of preterm delivery (34%) and LBW (32.4%) in adolescent pregnancies in our study population. The reduction in myometrial performance and difficulties in the process of delivery with aging may cause the increased risk of cesarean delivery with AMA pregnancies(19,20). We found a significant increase in cesarean delivery rate for AMA pregnancies compared with reproductive-age pregnancies, consistent with prior studies. The rates of cesarean delivery were similar between adolescent and AMA pregnancies.

Preeclampsia exposes a bimodal pattern with rises at the onset and end of reproductive ages^(20)^. Consistent with the literature, in our study preeclampsia was more frequent in the adolescent and AMA groups. Also, the number of eclampsia cases was similar between the three groups.

In a hospital-based study from Turkey, the proportions of preterm delivery in adolescent pregnancies were significantly higher than in reproductive-aged women^(21)^. AMA and adolescent pregnancies are independent risk factors for preterm delivery^(4,22)^. In our study, the highest preterm delivery rate was found in adolescent pregnancies. Contrary to prior studies, the preterm delivery rate was found similar between AMA and reproductive-age women. Additionally, the risk of spontaneous preterm delivery for adolescents was found 12.83-fold higher than in AMAs. However, iatrogenic preterm delivery rate was similar between these groups. We concluded that adolescent mothers who had immature uterine or cervical blood content may induce preterm delivery with increased prostaglandin output, and are more likely to give preterm delivery than advanced-age mothers.

The association between adolescent pregnancy and adverse obstetric outcomes were debated in the literature. Some authors indicated that LBW and preterm delivery rates increased in adolescent pregnancies compared with reproductive-age women, as a result of inadequate prenatal care. They also suggested that biologic immaturity was not the only reason for overall adverse obstetric outcomes^(23)^. In our study, several obstetric complications including placental abruption, PPROM, shoulder dystocia, retained placenta, and atonia were similar between the three groups. However, fetal distress and perineal injury rates were significantly higher in the adolescent group. With the exception of placenta previa, the rate of obstetric complications was similar in the adolescent and AMA groups. The placenta previa rate was higher in AMA due to the previous cesarean sections of the mothers.

Neonatal outcomes are directly affected by maternal status during pregnancy^(24)^. In the present study, babies of adolescent mothers were more likely to be admitted to the NICU, to have significantly lower birth weight, and lower 1st and 5th minute Apgar scores. Short-term neonatal complications, especially metabolic complications, were more common in adolescent pregnancies in the current study. Previous reports indicated that women with AMA had an increased risk of stillbirth^(25)^. The current study showed that AMA pregnancies had a significantly greater risk of stillbirth than reproductive-age women, consistent with previous studies. However, the most common risk for stillbirth was related to adolescent pregnancies according to this study. These results were likely to be caused by the higher preterm delivery rates of adolescent mothers.

There were several limitations in our study. This was a hospital-based, retrospective study performed in a low-income region of İstanbul. As such, these results cannot be considered to represent all pregnancy outcomes in Turkey. The strength of this study was the verification of the wide range of obstetric and neonatal outcomes between adolescent, AMA, and reproductive-age women, coupled with the experience of a single maternity hospital.

In conclusion, adolescent and AMA pregnancies should be defined as high-risk pregnancies. Our research indicated that healthcare providers such as obstetricians, midwives and family physicians should be alert in both adolescent and AMA pregnancies. Early, adequate, individualized, and multidisciplinary prenatal care might prevent perinatal complications among adolescent and AMA pregnancies. The biologic, ethnic, psychologic, social, and economic associations between these adverse effects could be clarified by further clinical and epidemiologic studies.

## Figures and Tables

**Table 1:**
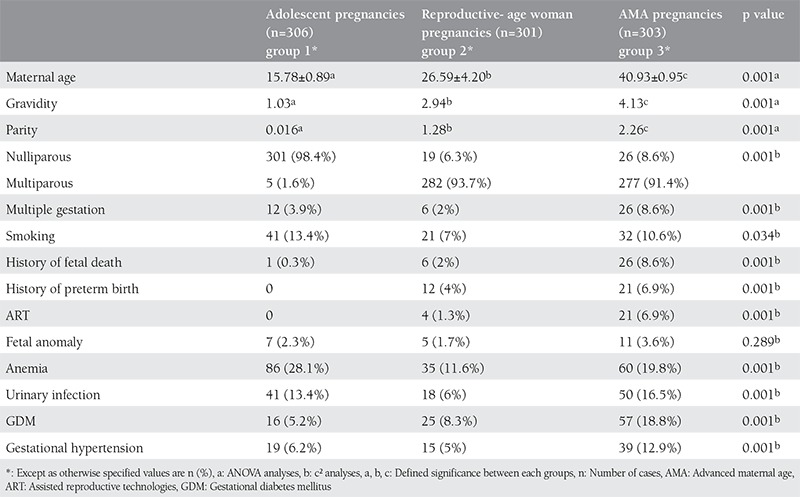
The clinical characteristics of adolescent, reproductive age and advanced maternal age pregnancies

**Table 2:**
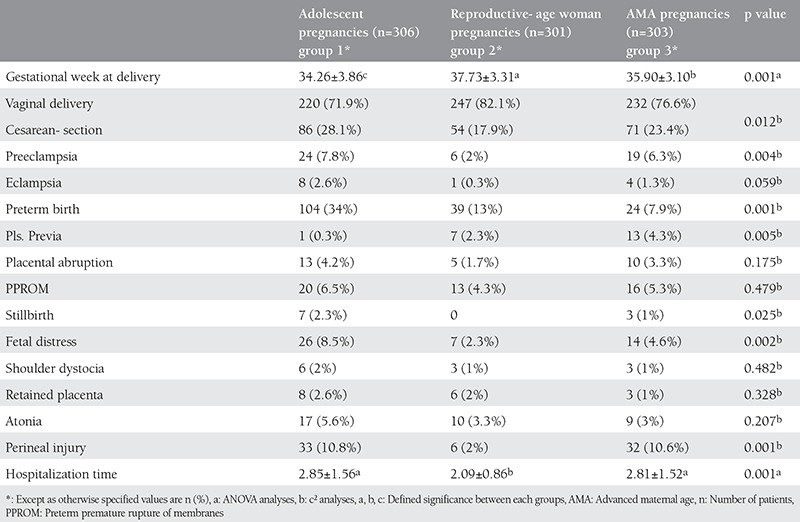
The obstetric outcomes of adolescent, reproductive age, and advanced maternal age pregnancies

**Table 3:**
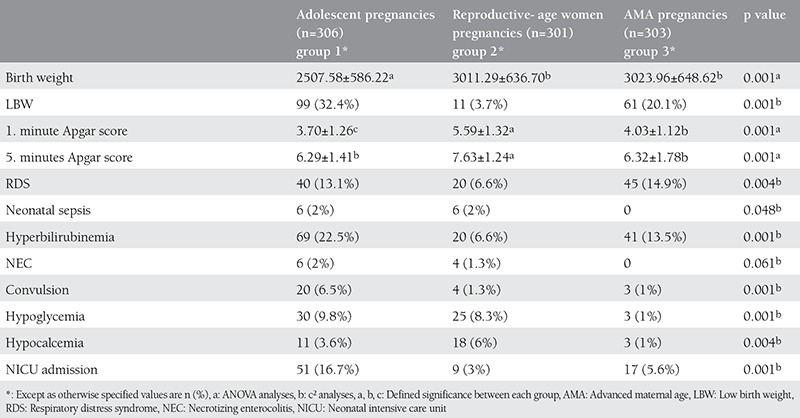
The short-term neonatal outcomes of adolescent, reproductive age, and advanced maternal age pregnancies
